# Differentiable multiphase flow model for physics-informed machine learning in reservoir pressure management

**DOI:** 10.1038/s41598-026-37063-3

**Published:** 2026-02-24

**Authors:** Harun Ur Rashid, Aleksandra Pachalieva, Daniel O’Malley

**Affiliations:** Earth and Environmental Sciences Division, Los Alamos National Laboratory, Los Alamos, NM 87545 USA

**Keywords:** Energy science and technology, Engineering, Mathematics and computing, Solid Earth sciences

## Abstract

Accurate subsurface reservoir pressure control is extremely challenging due to geological heterogeneity and multiphase fluid-flow dynamics. Predicting behavior in this setting relies on high-fidelity physics-based simulations that are computationally expensive. Yet, the uncertain, heterogeneous properties that control these flows make it necessary to perform many of these expensive simulations, which is often prohibitive. To address these challenges, we introduce a physics-informed machine learning workflow that couples a fully differentiable multiphase flow simulator, which is implemented in the DPFEHM framework with a convolutional neural network (CNN). The CNN learns to predict fluid extraction rates from heterogeneous permeability fields to enforce pressure limits at critical reservoir locations. By incorporating transient multiphase flow physics into the training process, our method enables more practical and accurate predictions for realistic injection-extraction scenarios compared to previous works. To speed up training, we pretrain the model on single-phase, steady-state simulations and then finetune it on full multiphase scenarios, which dramatically reduces the computational cost. We demonstrate that high-accuracy training can be achieved with fewer than three thousand full-physics multiphase flow simulations – compared to previous estimates requiring up to ten million. This drastic reduction in the number of simulations is achieved by leveraging transfer learning from much less expensive single phase simulations.

## Introduction

Reservoir pressure management refers to the control and maintenance of fluid pressure within a subsurface formation to optimize injection or extraction and prevent adverse effects caused by excessive or insufficient pressure. This is typically achieved through the injection or extraction of fluids to maintain a target pressure level within the reservoir^1^. The importance and application of pressure management span various fields, including carbon sequestration^2^, oil and gas production, subsurface gas storage^3^, geothermal energy extraction^4^, and wastewater disposal^5^.

Reservoir pressure management is essential since uncontrolled pressure can trigger seismicity and rock fracturing. Induced seismicity poses significant environmental and operational risks, including the potential for damaging earthquakes^6^. For example, large-scale wastewater re-injection in central Oklahoma led to unexpected seismic events^7,8^, while high-pressure water injection into the Basel geothermal reservoir in Switzerland induced seismicity that ultimately forced the cancellation of the project^9,10^. Preventing induced seismicity is also critical in subsurface gas storage (e.g., CO$_2$ and hydrogen), where long-term caprock integrity must be maintained to prevent leakage, which poses both environmental and economic risks^11^.

Most subsurface reservoirs are highly heterogeneous and uncertain. Their properties are often poorly characterized, fluids exhibit complex multiphase behavior, and the domains are typically large in scale^12–14^. A reliable pressure management tool must address these challenges and provide rapid predictions. However, the complexity of subsurface physics makes full-physics modeling expensive, and it is difficult to design a strategy that is both accurate and efficient enough for real-time decision-making.

Many high-fidelity simulation tools are available to predict subsurface pressure when reservoir properties are known. However, designing a robust pressure management strategy requires simulations of heterogeneous reservoir properties and multiphase fluid behaviors across a wide range of geological scenarios. In practice, this means running high-fidelity physics-based models on thousands of permeability realizations or more to account for the uncertainty and risk inherent to these problems. Unfortunately, traditional reservoir simulators struggle to handle the combined challenges of reservoir heterogeneity, multiphase flow, and uncertainty, driving computational cost so high that routine pressure modeling accounting for all these factors becomes untenable.

To over come the high computational cost, one potential solution is the use of surrogate machine learning (ML) models, which promise orders-of-magnitude speedups over physics-based simulators. Numerous ML studies in reservoir engineering have focused on applications such as, CO$_2$ storage^15–19^, enhanced oil recovery^20–24^, and geothermal energy^25,26^. However, these models are generally designed to predict system responses (e.g., pressure, saturation, production rates) rather than manage reservoir pressure in real time. Despite its critical role in preventing induced seismicity and caprock failure, ML in reservoir pressure management remains underdeveloped, highlighting the need for approaches that can learn effectively with sparse data while respecting underlying physics. While some of the CO$_2$ storage ML models cited above provide valuable predictive capabilities for known system response, their applicability to pressure management is limited. For pressure management application these data-driven models need high-quality datasets–such as well logs and production histories–that are rarely available for storage or disposal projects. These physics blind data-driven models may perform well in pressure management study when abundant data exist (as in mature oil and gas fields) but struggle in data-scarce CO$_2$ storage contexts, where only limited geological and operational data are available.

To overcome the physics blindness in data-driven models, several studies have embedded physical constraints (e.g. mass and momentum conservation) directly into the loss function of the neural network (NN), known as physics-informed neural networks (PINNs)^27,28^. PINNs are often insufficient in complex reservoir settings, resulting in overly simplified or approximate physics constraints. PINNs only softly constrain to the physics via the loss function and gradient descent, whereas traditional physics simulators have hard constraints that ensure the equations are accurately solved. The soft constraints often cause models to converge to incorrect solutions and produce flawed pressure management strategies.

A promising alternative to the limited physics constrained surrogate models is embedding a full-physics numerical simulator directly into the machine learning training loop, which promises more accurate, physics-consistent learning. This approach requires the simulator to be fully differentiable. However, traditional numerical models rely on finite-difference gradient estimates, which are incompatible with backpropagation algorithms used in gradient-based optimization^29–31^. To address this challenge, simulators can be developed using differentiable programming^32^ and automatic differentiation, enabling efficient and accurate gradient computation using the chain rule^33^. Differentiable programming enables the backpropagation that has traditionally been used with NNs to be fused with the adjoint methods that have been traditionally used in solving differential equations. Differentiable programming structures the simulator explicitly for integration into ML workflows and can be implemented using computational frameworks such as PyTorch^34^ and Julia^32^.

Despite recent advances in differentiable reservoir simulators^35,36^, their application to ML-driven pressure management problems remains limited. Pachalieva et al^37^. recently demonstrated the use of a fully differentiable single-phase steady-state flow model within a physics-informed ML framework, but to date no work has tackled multiphase flow models. Given that multiphase behavior dominates most reservoir operations^38,39^, integrating a differentiable transient multiphase simulator into the ML training loop is essential for enhancing accuracy in real-world pressure-control scenarios.

One of the major challenges of incorporating a multiphase flow simulator into a machine learning workflow is that many gradient descent steps, and if implemented in the obvious way, a proportional number of multiphase simulations would need to be performed. Harp et al^40^. identified that tens of millions of multiphase simulations would be required, which would be infeasible. Here, we circumvent this limitation by using transfer learning to dramatically reduce the computational cost of the training.

In this study, we introduce a ML workflow designed to determine the extraction rate required to maintain a prescribed pressure at critical locations in the reservoir during the injection period. Our training data consists of an ensemble of heterogeneous permeability fields, and we employ a transfer learning strategy, where we pretrain the model using a fully differentiable steady-state single-phase simulator and then finetune it with a transient multiphase flow simulator. The multiphase solver used in this study solves a two-phase, incompressible, and immiscible flow system. The key contributions of our work are (1) embedding a fully differentiable transient two-phase flow simulator in an ML training workflow; (2) employing transfer-learning strategies by pretraining on single-phase steady-state models before finetuning on two-phase flows.

This work is organized as follows. In the Methods section, we review the background physics and present the training workflow for reduced-order modeling. The Results section presents training and validation outcomes, demonstrates model accuracy through a case study and statistical analysis and present an application of the proposed workflow into a 3D muti-well system. In the Discussion section, we highlight the strengths and limitations of the proposed pressure management workflow and outline directions for future improvement. Finally, in the Conclusions, we summarize the novelty, strengths, applicability, and limitations of our approach.

## Methods

The primary objective of this study is to develop a surrogate model capable of determining the optimal fluid extraction rate required to manage overpressure within a subsurface reservoir. To achieve this, we train a NN using heterogeneous permeability fields as input to predict the optimal extraction rate. The predicted extraction rate is then passed through a full-physics, differentiable simulator, which evaluates the resulting pressure at a designated critical monitoring location. The simulated pressure is compared against a prescribed target value, and the resulting difference is used to compute the loss function for training the surrogate model.

In this section, we outline the complete methodology employed in this study. We begin by presenting the governing equations that underpin the physics-based simulator. This is followed by a description of the problem setup and the numerical solver used to compute the physical response of the system. We then illustrate the architecture of the NN model. Finally, we describe the training workflow in two stages: initially, the training loop incorporates only the two-phase simulator; subsequently, a transfer learning strategy is introduced, which uses a steady-state single-phase model in combination with a transient two-phase solver to accelerate training efficiency.

### Physics model

The full-physics simulator, central to our training loop, is governed by a set of flow equations designed to capture both steady-state single-phase and transient two-phase dynamics. Since our workflow employs a transfer learning approach that integrates both steady-state single-phase flow and transient two-phase flow, we provide a detailed description of the flow equations, their numerical discretizations, and the corresponding simulators for each set of equations. Both physics-based simulators are available as open-source tools within the DPFEHM GitHub repository^35^.

In the pretraining stage, we use a single-phase steady-state flow model, which assumes that pressure changes resulting from injection or extraction occur in a single-phase fluid and are independent of time^41^. In heterogeneous reservoirs, this leads to solving the following partial differential equation:

1 $$\begin{aligned} \nabla \cdot \left( K(x) \cdot \nabla p \right) = q, \end{aligned}$$

where *p* is the pressure, *K*(*x*) is the spatially varying permeability field, and *q* represents sources and sinks. The use of the steady-state single-phase flow equation enables rapid evaluation of how different extraction rates influence the pressure field, making it particularly well-suited for efficient pretraining. In this pretraining stage we use similar setup as pressure management example in DPFEHM GitHub repository^35^.

For the final training, the pressure change in the reservoir is calculated by considering transient two-phase fluid flow through a heterogeneous permeability field. Such a model involves solving the mass conservation equations for the phases present in the flow. Our differentiable simulator uses a sequential implicit pressure and explicit saturation (IMPES) scheme for incompressible and immiscible two-phase flow, as described by Aarnes et al^42^.. As in the Aarnes et al^42^., we neglect gravity and capillary forces for simplicity. Based on these assumptions, we solve the following governing equations for pressure (*p*) and saturation (*s*) respectively:

2 $$\begin{aligned}&\text {Pressure equation:}\quad \;\; -\nabla \cdot K(x)\lambda (s)\nabla p = q, \end{aligned}$$

3 $$\begin{aligned}&\text {Saturation equation:}\quad \phi \frac{\partial s}{\partial t}+\nabla \cdot \left( f(s)v\right) = \frac{q_w}{\rho _w}. \end{aligned}$$

Here, $\lambda$ in pressure equation is the total mobility of the phases and is a function of saturation. In the saturation equation the term $\phi$ represents the porosity of the medium, *t* is time, *f*(*s*)*v* represents the viscous force, where *v* is the velocity of the phase and *f*(*s*) is fractional flow function. On the right side of the saturation equation, $q_w$ is the wetting phase injection rate and $\rho _w$ represents the density of wetting phase. The term $\frac{q_w}{\rho _w}$ represents the source. In our simulator, we only inject the wetting phase. Therefore, the source term can be modified as follows:

4 $$\begin{aligned} \frac{q_w}{\rho _w}=max(q,0)+f(s)min (q,0). \end{aligned}$$

In our model, we calculate the mobility of wetting and non-wetting phase using the following analytical expression:

5 $$\begin{aligned} \lambda _w(s)=\frac{(s^*)^2}{\mu _w}. \end{aligned}$$

6 $$\begin{aligned} \lambda _{nw}(s)=\frac{(1-s^*)^2}{\mu _{nw}}, \end{aligned}$$

with $s^*$ defined as follows:

7 $$\begin{aligned} s^*=\frac{s-s_{wc}}{1-s_{nwr}-s_{wc}}. \end{aligned}$$

Here, the term $s_{nwr}$ represents the irreducible saturation for the non-wetting phase, which is the minimum amount of fluid that remain in the reservoir despite the displacement, and $s_{wc}$ is the connate saturation for the wetting phase, which is the minimum fluid saturation exist naturally in the pores and generally is immobile due to high capillary pressure.

Finally we can discretize the saturation equation using finite-volume scheme:

8 $$\begin{aligned} s_{i}^{n+1}=s_i^n+(\delta _x^t)_{i}\left( max(q_i,0)-\sum _{j}f(s^n)_{ij}v_{ij}+f(s^{n}_{i}) min(q_{i},0)\right) , \end{aligned}$$

where the superscript, $n+1$ represent the value of the variable in the current time step, and *n* represents the value in the previous time step. We calculate the term $(\delta _x^t)_{i}$ based on the CFL (Courant-Friedrichs-Lewy) time step condition^43^, which determines the maximum allowable timestep that ensures numerical stability and prevents non-physical oscillations or instabilities during simulation.

To solve the single-phase and two-phase flow equations, we utilize the DPFEHM framework^35^. DPFEHM is an automatically differentiable Julia package that implements standard two-point flux approximation and finite volume methods for numerical discretization. Its built-in support for automatic differentiation enables seamless integration of the full-physics models into our machine learning workflow, allowing for efficient backpropagation through the simulation process.

**Fig. 1:**
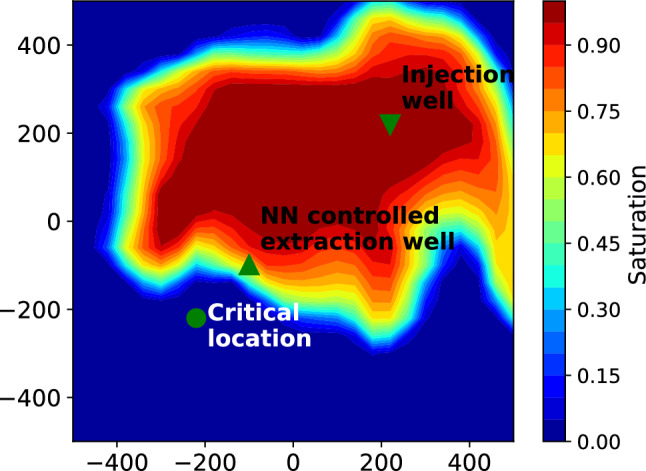
Simulation domain for the pressure management workflow, showing well locations and the saturation front of the injected fluid in the background. The NN-controlled extraction well withdraws a portion of the injected fluid to maintain the prescribed pressure at the critical location.

Figure 1 illustrates the layout of the simulation domain. The simulation is conducted on a 2D domain measuring 1000 by 1000 meters. The setup includes one injection and one extraction well, marked with a downward-pointing and upward-pointing triangle, respectively. Fluid is injected at a constant rate of 0.031688 $m^3/s$ (approx. 1 Mt/year) at the injection location. The critical location is marked by a circle. Our pressure management workflow aims to maintain a prescribed pressure at this location throughout the injection period, which is ensured by the optimum extraction rate at the extraction well. The extraction well is strategically positioned between the injection and the critical locations, and is closer to the latter to maximize its control over the pressure in the target region. For this study, we used the simulation and geostatistical parameters listed in Table 1.

**Table 1:** Parameters used in physics model.

Parameters	Value	Unit
Boundary condition	Dirichlet pressure	-
Boundary pressure value	0	Pa
Irreducible fluid saturation	0	-
Connate fluid saturation	0	-
Porosity	1	-
Viscosity of wetting phase	1	Pa-s
Viscosity of non-wetting phase	1	Pa-s
Variogram (Covariance) type	Matern, exponential	-
Marginal standard deviation	1	-
Correlation length	50	m
Sampling Method	Karhunen-Loève (KL)	-
Number of KL modes	200	-

### Neural network model

With the physics solver in place, we define NN architecture of our surrogate model. Since the input is a two-dimensional, spatially varying permeability field, we employ a convolutional neural network (CNN), which is well-suited for processing structured spatial data. Specifically, our model builds upon the classic LeNet-5 architecture proposed by LeCun et al^44^., which combines convolutional encoding layers with fully connected dense layers to process spatially structured inputs. In this study, we adopt a slightly modified version of LeNet-5, closely following the physics-informed architecture introduced by Pachalieva et al^37^.. Because our physics simulator is more complex than the steady-state simulator used by Pachalieva et al^37^., we initially tested a deeper network architecture based on VGG16^45^, which includes twelve convolutional layers and three dense layers. However, this more complex model performed worse than the simpler LeNet-5 architecture. The likely reason is that the larger network was not well suited to our relatively simple pretraining physics model. In general, deeper networks create more intricate response surfaces, increasing the risk of the optimizer becoming trapped in local minima. In contrast, LeNet-5 provides a good balance between representational power and training stability. Identifying the optimal network architecture for physics-informed learning remains an open and promising direction for future research.

Our CNN architecture consists of two convolutional layers, each followed by max-pooling subsampling layers, a flattening step, and a fully connected dense block. The convolutional layers apply $5\times 5$ kernels to extract spatial features from the input permeability field. The first layer outputs 6 feature maps, while the second produces 16. Each convolutional layer is followed by a max-pooling layers with a stride of 2, which reduces the spatial resolution by a factor of 4 across the two layers, thereby enhancing computational efficiency. The output of the convolutional block, originally in a four-dimensional tensor format, is flattened into a two-dimensional vector to interface with the subsequent dense block. This dense block consists of three fully connected layers containing 120, 84, and 1 neurons, respectively. Rectified Linear Unit (ReLU) activation functions are applied to all hidden layers, defined as $\sigma (x) = \max (0, x)$, where *x* is the input and $\sigma (x)$ is the activated output. The final layer yields a single scalar value, representing the predicted extraction rate. The NN parameters used for this study are listed in Table 2, and the modified CNN architecture is summarized below:
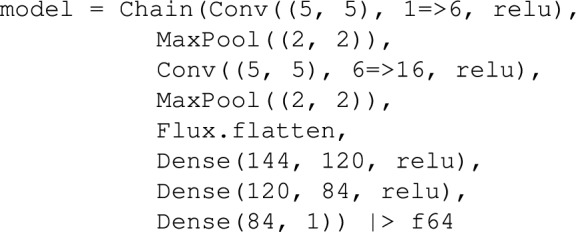

**Table 2:** Parameters used in training NN model.

Parameters	Value
Learning rate	$10^{-4}$
Batch size	40
Samples per epoch	200
Optimizer	ADAM

The permeability samples used for training are randomly generated and vary in each epoch, thus the NN model never sees the same permeability field during training. For validation, 200 samples are fixed throughout the process, while the 10,000 test samples – used to evaluate the model’s performance – are also randomly generated.

### Quantification of prediction errors

To evaluate the model performance and quantify prediction errors, we define a loss function based on the difference between the simulated and prescribed pressures at the critical location. The loss function is given by:

9 $$\begin{aligned} \mathcal {L}=\sum _{i}^{N_b}\sum _{j}^{N_s}\left[ \Delta p \left( q_{nn}(\theta ,k_j(x)),k_j(x)\right) -\Delta p^{target}\right] ^2, \end{aligned}$$

where $N_b$ is the number of training batches, $N_s$ is the number of samples per batch, $p^{target}$ is the prescribed targeted pressure, and $\Delta p$ is the simulated pressure response based on the the predicted extraction rate, $q_{nn}(\theta ,k_j(x))$ generated by the NN model. The NN model predicts the extraction rate based on model parameters $\theta$ and the permeability field $k_j(x)$. During training, the NN minimizes the loss function by updating its parameters through backpropagation. This iterative process drives the model toward an optimal estimation of the extraction rate, which minimizes pressure error at the critical location.

To evaluate the model’s performance, we compute the root mean squared error (RMSE), defined as:

10 $$\begin{aligned} RMSE(\theta )=\sqrt{\frac{\mathcal {L}(\theta )}{N_b N_s}}. \end{aligned}$$

The RMSE is a standard metric for evaluating model accuracy across all training samples, enabling consistent comparison and monitoring of convergence during the training process.

### Pressure management workflow

**Fig. 2:**
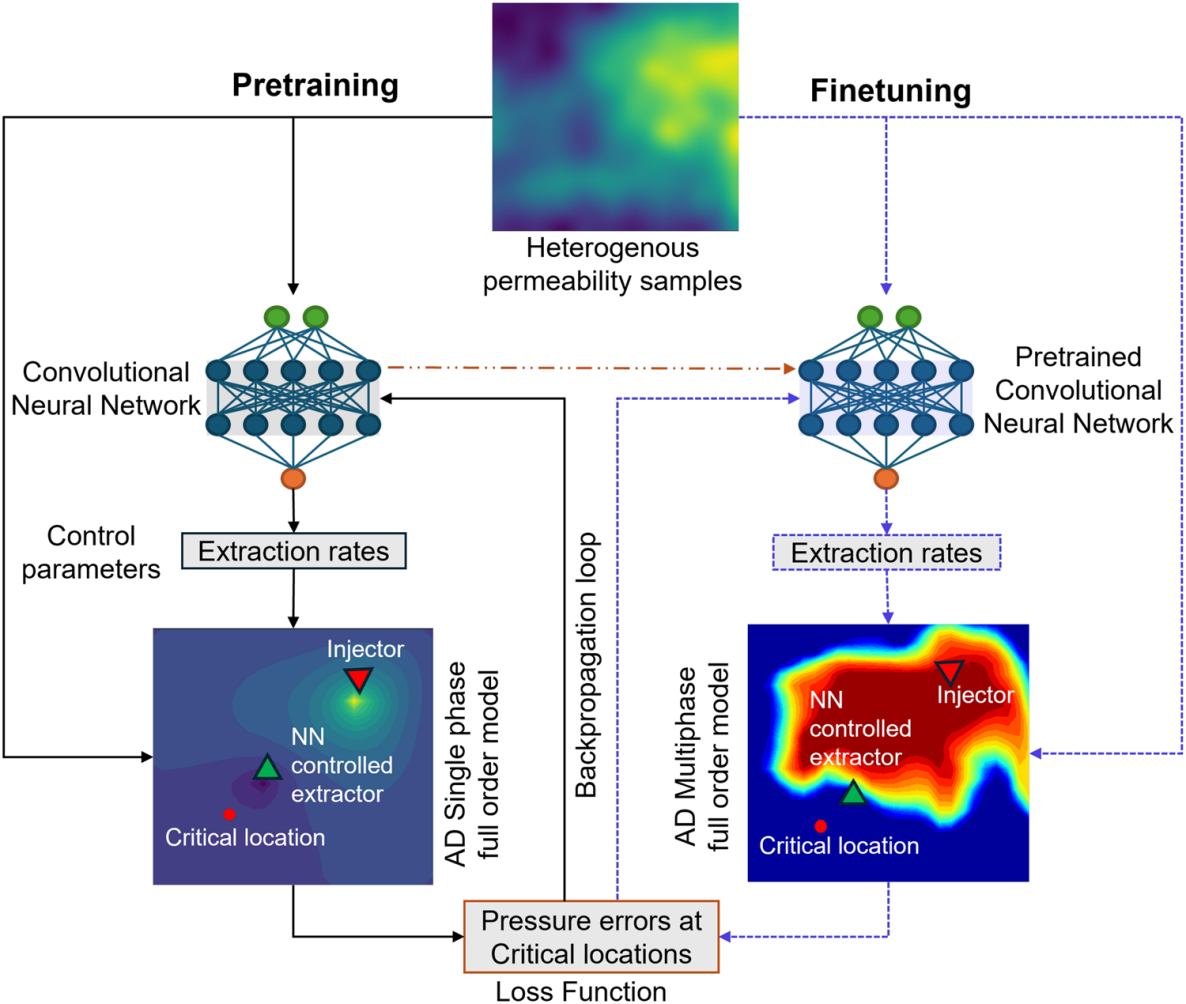
Full training workflow for the pressure management model using a transfer learning approach, which enables efficient incorporation of complex physics. The model is initially pretrained using fast single-phase steady-state simulations, and then finetuned with transient two-phase simulations during the final training stage.

Having set up both the physics-based simulator and the NN model, we now integrate them into a unified training workflow designed for pressure management in the subsurface. This workflow, illustrated in Fig. 2, follows a two-stage approach consisting of pretraining and finetuning stages. Each stage begins by sampling random realizations of heterogeneous permeability fields and specifying a target pressure at a critical reservoir location. Each permeability realization is passed through the NN, which predicts the corresponding extraction rate. The predicted extraction rate is then used as input to the full-physics simulator, which computes the resulting pressure distribution. The pressure at the critical location is compared to the prescribed target, and the difference, referred to as the pressure error, is used to perform backpropagation. This error signal guides gradient-based optimization to iteratively improve the NN parameters.

A similar training workflow has been explored in previous studies by Harp et al^40^., Srinivasan et al^46^., and Pachalieva et al^37^.. However, the approaches by Harp and Srinivasan relied on simplified, differentiable analytical solutions, while Pachalieva et al. focused exclusively on single-phase steady-state flow. In contrast, our work is the first to integrate a fully differentiable transient two-phase flow simulator as part of the training loop, enabling more realistic and physically comprehensive modeling of subsurface pressure dynamics.

We employ a transfer learning strategy in our training workflow to mitigate the high computational cost associated with two-phase flow simulations. In the context of deep learning, transfer learning is a training strategy which improves a model’s performance on a target domain by leveraging knowledge learned in a related source domain^47^. This allows the model to fist capture generalizable patterns and then finetune it’s predictions using more challenging scenarios^48^.

In our transfer learning workflow, we first pretrain the CNN using a single-phase steady-state flow model to accelerate convergence and reduce computational cost. The simulation setup for this stage is illustrated in Fig. 1. The primary objective during pretraining is to enable the model to learn the mapping from heterogeneous permeability fields to extraction rates that maintain the prescribed pressure at a critical location, using the simplified single-phase steady-state formulation. Once the model achieves satisfactory accuracy in this setting, we transition to the more complex two-phase flow regime by replacing the single-phase simulator with the transient two-phase flow model. This sequential training strategy is illustrated in Fig. 2, where the left panel represents the pretraining phase using steady-state single-phase flow, and the right panel depicts the final training phase involving time-dependent two-phase flow.

In both the single-phase and two-phase settings, the NN model predicts the extraction rate based on the input permeability field. Due to the consistency in simulation setup and input data structure across both phases and the high-level physical similarity, the transition from the single-phase to the two-phase model does not degrade the model’s predictive performance too badly. Additionally, since the pressure error is used as the training loss, which does not differ drastically between steady-state and transient simulations, the model is able to maintain its accuracy during the transition.

However, it is important to note that this transferability may not extend as well to other flow variables such as saturation in a two-phase flow. In such cases, the simplifications inherent in single-phase pretraining may not be sufficient to enable predictive accuracy during the transition to a two-phase framework.

The use of differentiable programming provides our approach a clear advantage over traditional approaches which use finite difference method to compute gradient of parameters. Unlike finite difference schemes, which estimate sensitivities through repeated perturbations of parameters, differentiable programming breaks the entire physics-based simulator into a composition of elementary differentiable operations and uses chain rules for calculating complex derivatives. This makes the simulator compatible with machine learning frameworks to be incorporated in the training loop. Differentiable programming is particularly efficient when the number of model parameters is large, since the computational cost of forward and backward passes does not depend on the dimensionality of the parameter space. In our workflow, the neural network predicts the extraction rate, which is then passed to the physics solver to compute the pressure distribution across the domain. The resulting pressure at the critical location is compared with the prescribed target pressure, and the loss function is defined as the pressure mismatch. During backpropagation, the gradient of the loss with respect to the neural network parameters is computed by differentiating through both the physics solver and the NN using the chain rule. This backward pass requires only the solution of the system of equations, enabling efficient training while preserving the underlying governing physics.

## Results

In this section, we first present training results where we trained the NN from scratch using the transient two-phase flow solver to demonstrate the challenges of training directly on complex physics. Next, we show the training and validation losses obtained using the transfer learning workflow, which reflect the model’s ability to manage the pressure. Then, we demonstrate the model’s effectiveness through a case study to confirm its ability to control pressure at the critical location. Finally, we evaluate the model’s performance on a set of randomly generated permeability fields, comparing the predicted extraction rates and the resulting pressure outcomes.

Before applying the transfer learning strategy, we trained the NN from scratch using data generated by the transient two-phase flow solver. This was done to illustrate the difficulties associated with learning complex physical dynamics without prior knowledge. This baseline workflow mirrors the finetuning stage depicted in Fig. 2, with the key difference that the NN is initialized with random weights. To ensure that the model can capture meaningful variations in the pressure field, we simulated one year of fluid flow. The simulated pressure at the end of the run was compared to the target pressure, and the resulting difference was used to compute the training loss.

**Fig. 3:**
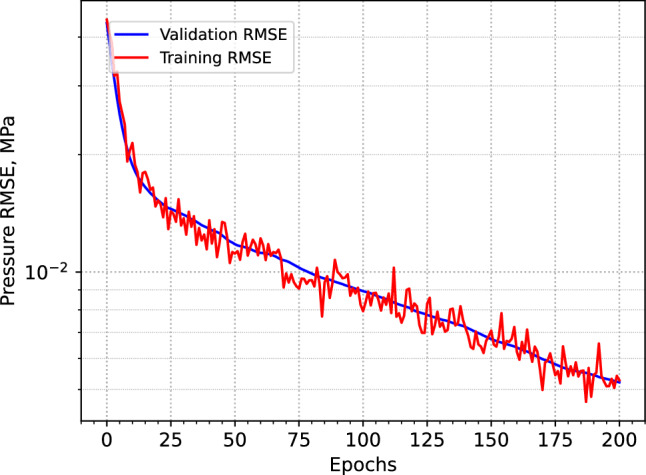
Training and validation RMSEs from the initial training using only the two-phase solver. The high computational cost of incorporating complex physics makes this approach challenging, highlighting the need for more efficient learning strategies.

The baseline training results confirm that the training NN models using two-phase flow simulations over extended run-time is computationally expensive. As shown in Fig. 3, while the model begins to learn meaningful patterns, the convergence remains notably slow. To accelerate the process, we parallelized the training using Julia’s Message Passing Interface (MPI) framework, employing 40 processors in parallel. Despite this optimization, simulating one-year injection scenario over 200 training epochs required approximately 11 CPU hours with an AMD EPYC 7702P 64-Core processor.

Later in this section, we show that applying transfer learning significantly improves model accuracy, while reducing the computational cost by an order of magnitude. These results highlight the inefficiency of relying solely on two-phase simulations over extended run-time during the early stages of training.

**Fig. 4:**
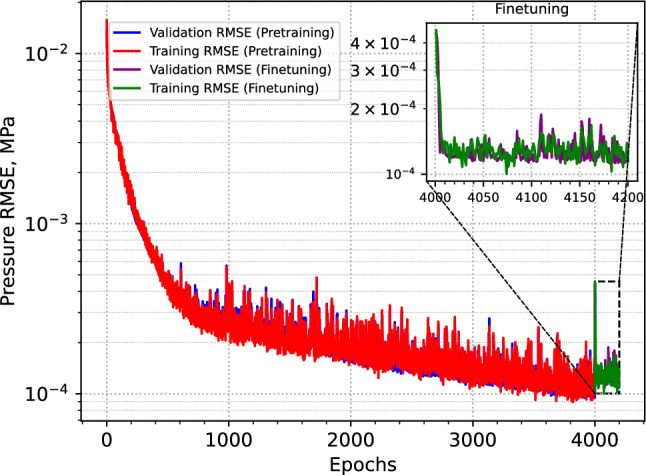
Training and validation RMSEs over the course of transfer learning. The smooth convergence of both curves indicates a stable knowledge transfer, with error approaching a non significant value. By the end of training, the pressure error falls to as low as 0.0001 MPa.

Next, we used our transfer learning workflow to train the NN model, which determine the extraction rate at the extraction well. Figure 4 presents the RMSE observed during both the training and validation stages of the transfer learning workflow. The red and blue lines correspond to the validation and training errors during the pretraining phase, respectively, while the yellow and green lines represent the validation and training errors during the final two-phase training phase.

As illustrated in the figure, transitioning from the single-phase steady-state simulator to the transient two-phase simulator only marginally degrades the model’s performance. Both training and validation errors continue to decline steadily throughout the training process, indicating a smooth and effective transfer of learned representations between physical models. A closer examination of the zoomed-in final training results reveals that the model achieves its minimum error within just 20 epochs after the transition.

The total training time was approximately 5 CPU hours, including 1 hour for the pretraining phase and 4 hours for finetuning with the two-phase flow solver – highlighting a significant gain in training efficiency. Most of the loss reduction during finetuning occurs within the first 13 epochs, requiring only about 0.3 CPU hours. If training is halted at that point, the total training time drops to just 1.3 CPU hours. The results indicate that incorporating transfer learning allows the model to maintain high accuracy while reducing overall computational cost by an order of magnitude.

While the initialization of a model’s weights can influence training cost, it is less significant in our transfer learning workflow. In this workflow, the pretraining stage initializes the model weights for finetuning, and its computational cost is relatively small. We tested multiple weight initialization cases to evaluate their effect on model convergence. Regardless of the initialization used, the pretrained model always reached the same target accuracy in nearly the same number of epochs. The difference is negligible, especially since the pretraining cost is small compared to the finetuning phase. This indicates that the choice of initialization weights does not significantly affect the finetuning process, as it begins with nearly identical weights regardless of initialization. Consequently, the overall training cost–dominated by the finetuning stage, remains effectively the same.

As shown by the training and validation errors in Fig. 4, the pretrained model rapidly reaches a high level of accuracy, with errors dropping well below 0.0001 MPa. This level of precision indicates that the model has undergone sufficient training to accurately predict optimized extraction rates for a wide range of heterogeneous permeability fields. To further test this, we evaluate the model’s performance on a set of randomly generated permeability realizations and analyze the resulting pressure responses using the predicted extraction rates. The testing procedure is described in detail in the following evaluation section.

### Evaluation of pressure management workflow

After training the NN model, we employ it to predict suitable extraction rates and subsequently calculate the resulting overpressure at the critical location using a full physics two-phase simulation. This process allows us to evaluate the effectiveness of the NN model in accurately determining extraction rates to maintain the desired pressure near the critical location.

**Fig. 5:**
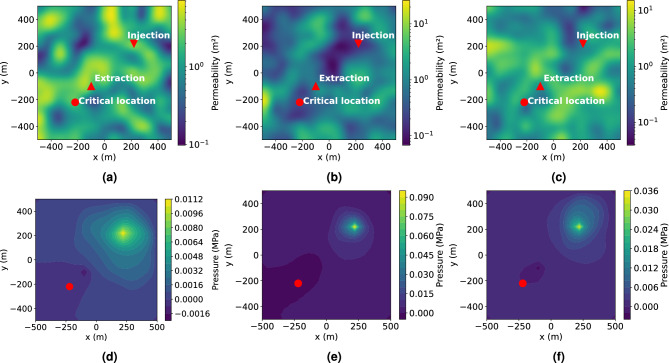
Evaluation of the trained model using three test cases. (**a**,**b**,**c**), Input permeability field for the trained model. (**d**,**e**,**f**) corresponding pressure field from full physics simulation with predicted extraction rate.

Initially, we demonstrate this approach using a few sample permeability fields, illustrated in Fig.e 5, where Figure 5a, b, and c show the random permeability samples and the corresponding pressure field generated by full physics simulation is shown in Fig. 5d, e and f. In this Figures, a negative pressure region is clearly observed around the extraction well, indicative of fluid withdrawal. Moreover, the pressure reaches zero around the critical location, which is highlighted by a red dots in the figures, matching the prescribed pressure for the location. This result confirms the NN model’s capability to successfully predict an extraction rate that maintains the specified pressure at a critical reservoir location. In these examples, the permeability values span approximately three orders of magnitude. The resulting pressure distributions also exhibit high sensitivity to permeability variations. For instance, the case shown in Fig. 5e has a maximum reservoir pressure of 0.09 MPa, which is eight times higher than that of the case in Fig. 5d.

**Fig. 6:**
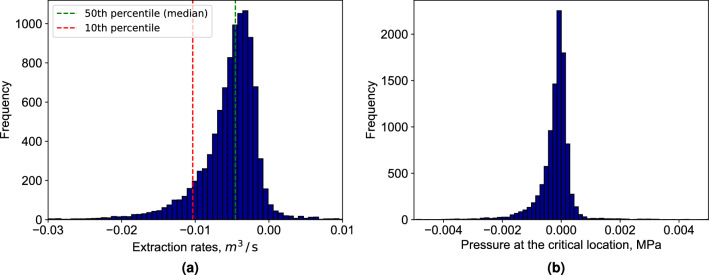
(**a**) Extraction rates in cubic meters per second [$m^3/s$]; (**b**) resulting pressure at the critical location in mega pascals [*MPa*]. For input of 10,000 random permeability fields the NN model predicts extraction rates, which maintain zero or near zero pressure at the critical location for most the cases. It confirms that the trained model can be used to handle uncertainty and heterogeneity for pressure management in a field.

Next, we analyze the distribution of the predicted extraction rates and the corresponding overpressure at the critical location. To generate these distributions, we pass 10,000 randomly generated heterogeneous permeability fields (training set from Table 2) through our trained NN model. For each case, we compute the resulting overpressure using the full physics simulation. The outcomes are presented in Fig. 6, where the predicted extraction rates are shown in Fig. 6a, and the corresponding overpressure values are illustrated in Fig. 6b. The distribution of extraction rates is notably skewed, with 90% of the samples requiring an extraction rate below 0.01 m^3^/s (equivalent to 0.32 Mt/year) to maintain the target pressure. The average extraction rate is 0.0046 m^3^/s, or 0.15 Mt/year. Compared to the injection rate of approximately 1 Mt/year, the average extraction rate is about 15%, which means for 50% of samples we need to extract 15% or less of the injected fluid. Meanwhile, the overpressure distribution reveals only minor deviations from the prescribed pressure of zero at the critical location for most of the samples. The negative pressure values at the critical location is related to the pressure target we set. For this study, the target pressure is prescribed as zero MPa. During optimization, the neural network minimizes deviations from this target by adjusting the extraction rate. Because the pressure field is continuous, local drawdown near the extraction well can occasionally produce slightly negative pressures. Occasional small positive extraction rates were also observed in some cases. These arise because the output layer of the neural network is unconstrained, and the pressure control objective is nearly symmetric around zero.

These results ensure the model’s capability to consistently predict extraction rates that maintain the target pressure at critical points within the reservoirs with random heterogeneous permeability field. The pressure evaluation shows improved accuracy compared to past work that handled only single phase flow^37^.

### Model applicability and reusability

In many reservoir management workflows, decision-making may require only a small number of simulations. In such cases, the cost of training a surrogate model like ours may not be justified, and direct numerical simulations may remain a more practical and efficient choice. However, the cost of pretraining and finetuning in the proposed workflow becomes worthwhile in applications that require repeated evaluations such as uncertainty quantification, optimization, or sensitivity analysis. In these applications, when reservoir heterogeneity is uncertain, making reliable decisions based on only tens or hundreds of simulations is not possible. It is often necessary to estimate the distribution of possible outcomes by running thousands of realizations with different permeability fields. For applications like the reservoir pressure management problem presented in this study, the optimal extraction rate would need to be computed by solving a per-sample optimization problem using the full simulator. For systems with uncertain heterogeneity, this would require performing such optimizations for thousands of realizations, which is computationally impractical. In that case, the cost of training a surrogate model, as demonstrated in this work, becomes highly beneficial.

**Fig. 7:**
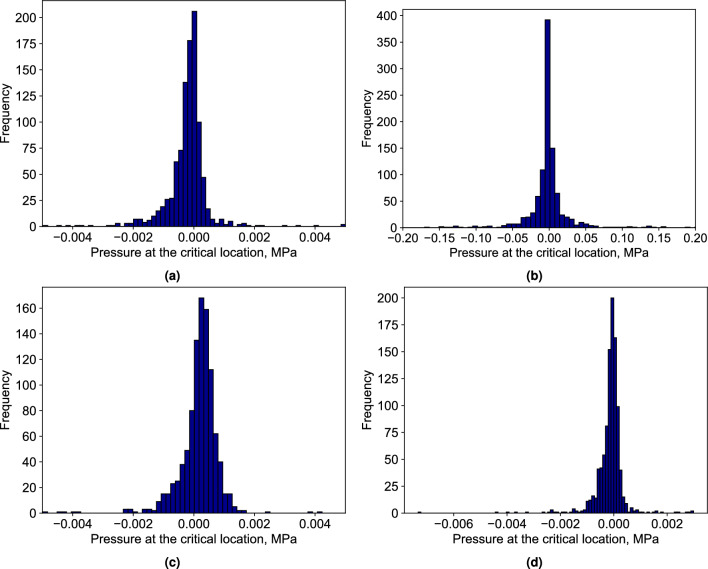
Model’s performance in maintaining pressure at the critical location under modified reservoir conditions (**a**) Pressure distribution when we change the correlation length to 100 m; (**b**) pressure distribution when change the standard deviation from 1 to 2; (**c**) pressure distribution when change the extraction well location; (**d**) pressure distribution when change the fluid viscosity from 1 to 0.8 Pa-s.

In field applications, the proposed workflow is suitable for analyzing geologic storage sites where only limited information about formation heterogeneity is available. The workflow can be applied to feasibility studies, uncertainty quantification, or the optimization of operational parameters in such formations. To implement it, a training dataset and corresponding physics setup must first be generated based on the available system information. Using the known petrophysical data, one can define the parameters of the Gaussian random field–such as the standard deviation and correlation length–which are then used to generate random permeability realizations. Geological information on faults or damaged zones within the reservoir can also guide the selection of critical monitoring locations and well placements. Once the training dataset and physics setup are established, the workflow can be used to train a surrogate model for the intended application.

Next, we investigate the model’s performance in maintaining pressure at the critical location under modified reservoir conditions. Specifically, we analyze the sensitivity of the model to changes in (i) the correlation length (ii), and standard deviation of the Gaussian random field, (iii) well locations, and (iv) fluid viscosity. For each case, 10,000 randomly generated permeability samples were used to evaluate the model’s performance under these conditions.

In Fig. 7a, shows the pressure distribution at the critical location when we change the correlation length of the Gaussian random field from 50 m to 100 m. Based on the distribution, we can see that the model is able to maintain the target pressure at the critical location under this condition. However, the pressure distribution becomes slightly broader than the original distribution which uses 50 m correlation length. As we do not see any significant change in the distribution and error range, we can come to the conclusion that changes in correlation length do not significantly affect the model’s performance and we do not need to train a new model for this change

In Fig. 7b, we investigate the effect of changing the standard deviation of the Gaussian field, which controls the degree of heterogeneity. We increase the standard deviation from 1 to 2, which results in larger pressure deviations at the critical location, suggesting that the trained model produces higher prediction errors for more heterogeneous systems. The pressure distribution shows a larger error range, with significantly higher error values. This implies that, for changing the standard deviation of the distribution, the model would require retraining to maintain accuracy.

Next, we examine the impact of shifting the well locations. We moved the extraction well location and the corresponding critical point by seven grid blocks to the right. As shown in Fig. 7c, this modification does not impact pressure errors significantly, indicating that the model generalizes well to small changes in well locations. We do not need to retrain the model in this case

Finally, we assess the influence of fluid properties by reducing the viscosity of both phases from 1 Pa-s to 0.8 Pa-s. The resulting pressures at the critical location are shown in Fig. 7d. While the overall pressure distribution becomes slightly broader, most samples still remain within the prescribed pressure limits, showing only minor deviations. This indicates that slight change in fluid viscosity, does not impact the model’s performance.

Although many other parameters and broader data ranges could be explored to evaluate the full reusability of the trained model, such an extensive study is beyond the scope of the present study. Here, our goal is to demonstrate that certain parameter modifications do not significantly affect model performance, while others–such as increased heterogeneity–do. Therefore, assessing the model’s reusability should be an essential step before applying it to new or modified reservoir settings.

### Application of the pressure management workflow in a 3D multi-well reservoir

In this section, we extend our workflow to demonstrate its application for pressure management in a heterogeneous three-dimensional (3D) reservoir containing multiple injection and extraction wells. The overall training procedure, loss formulation, and physics-based simulator remain unchanged; only the dimensions of the input and output layers of the CNN are modified to accommodate the 3D configuration. The modified CNN architecture is shown below:
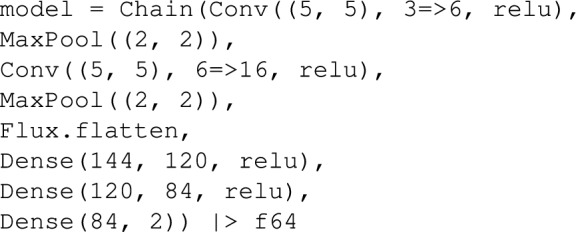


To ensure sufficient learning for this more complex setup, the total number of epochs during pretraining was increased to 6000, while finetuning was performed for 200 epochs, consistent with the 2D case. The total training time for the pretraining stage was approximately 2 CPU hours using the same computational resources as in the 2D setup. The finetuning stage required about 26 CPU hours on the same machine. Similar to the 2D case, the finetuning process was parallelized using MPI.

**Fig. 8:**
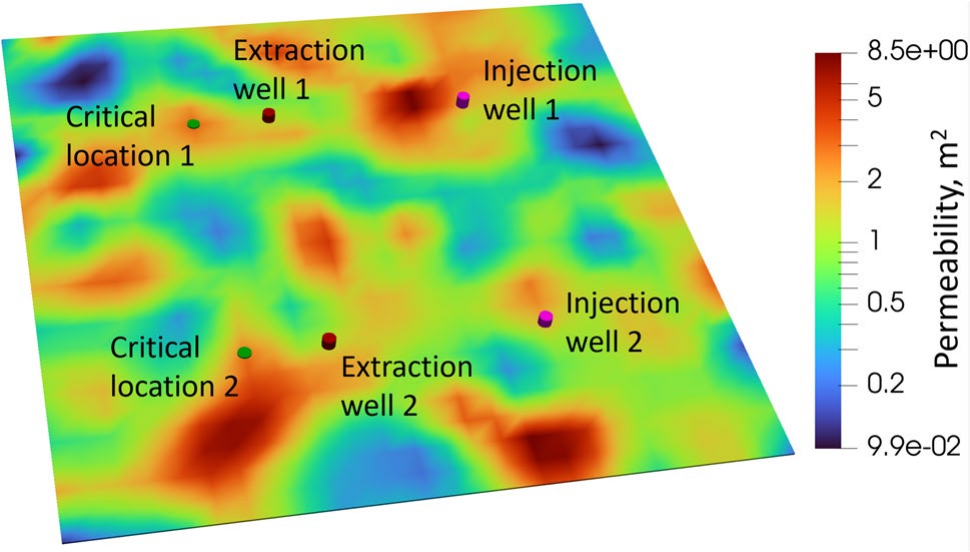
3D multi-well system for the pressure management workflow. The figure shows two injection wells, two extraction wells, and two critical monitoring locations. The CNN model predicts extraction rates that maintain the pressure within the prescribed limits at these critical locations.

**Fig. 9:**
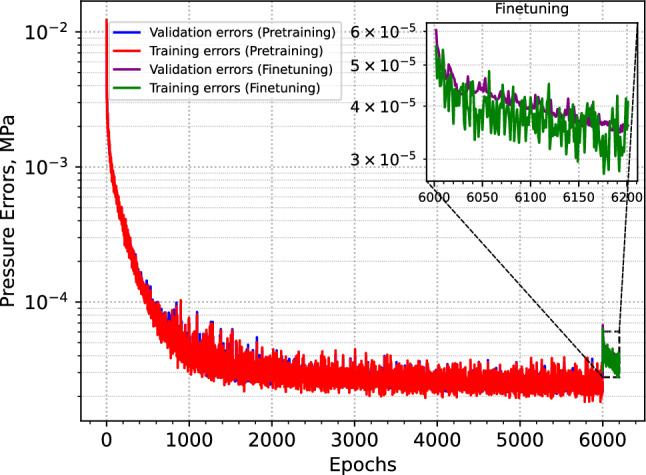
Training and validation RMSEs during transfer learning for the 3D multi-well system. The smooth convergence of both curves indicates stable knowledge transfer, with the error approaching a negligible value. By the end of training, the pressure error reduces to as low as 0.00002 MPa.

The 3D reservoir settings used for this problem is illustrated in Fig. 8. In this configuration, we consider a three-layered 3D reservoir with two injection wells and two extraction wells. We set two critical locations for the this reservoir. Like the 2D case the extraction wells lies between the injection wells and the critical locations and closer to the critical locations. The CNN model predicts extraction rates for the extraction wells to maintain the reservoir pressure within the prescribed limits at the critical locations. The physical dimensions of the reservoir in the *x* and *y* directions remain the same as in the 2D case, while in the *z* direction, we used six meter reservoir depth and discretized it into three layers. This setup represents a more complex and realistic scenario compared to the previously discussed two-dimensional case with a single pair of injection and extraction wells.

Figure 9 shows the training and validation errors for the 3D multi-well system. The blue and red curves represent the training and validation errors for the pretraining stage, while green and purple correspond to the finetuning stage training and validation errors respectively. Similar to the 2D case, the pretraining of the model with single phase achieves an effective learning goal. When we switch to the two-phase solver in finetuning, after an increase in the loss, the model quickly adjust to the changed physics and rapidly reaches the same level of error as the pretraining within a few epochs. In this case, both the training and validation errors are approximately an order of magnitude lower than those in the 2D case. This is primarily due to the larger reservoir volume, which leads to lower overall injection pressures.

**Fig. 10:**
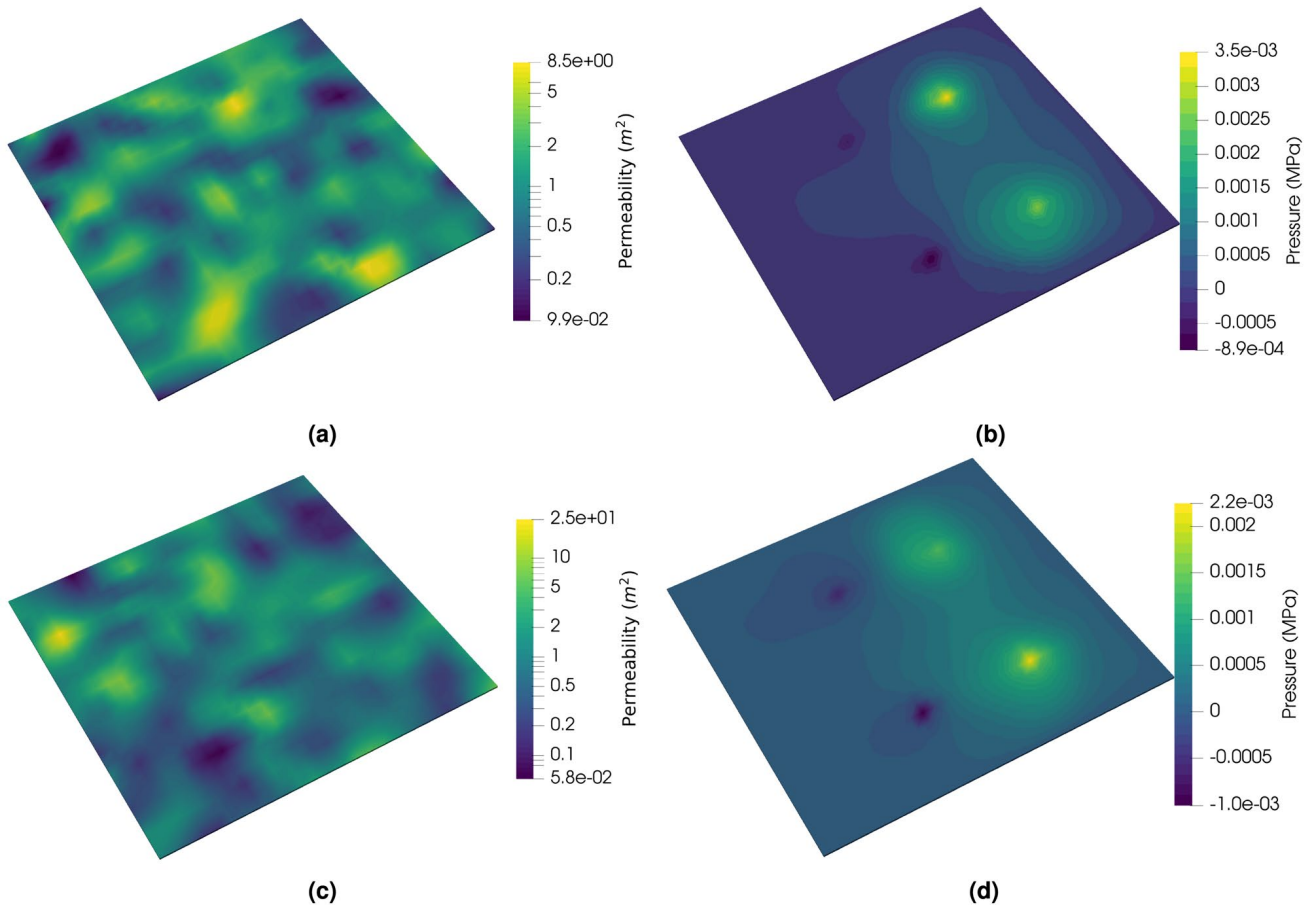
Two test cases for the 3D multi-well system showing random permeability samples and the corresponding pressure profiles from the full-physics simulations using the trained CNN model.

**Fig. 11:**
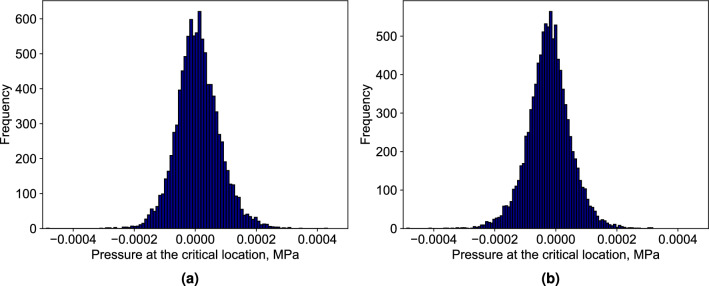
(**a**) Pressure distribution at critical location 1; (**b**) Pressure distribution at critical location 2. The trained neural network predicts extraction rates that maintain zero or near-zero pressure at the critical points in most cases, confirming its effectiveness for pressure management in heterogeneous multi-well 3D reservoirs.

In Fig. 10, we present several representative test cases. For each case, a randomly generated permeability field is selected, and the corresponding pressure profile from the full-physics simulation is plotted using the extraction rates predicted by the CNN model. In all cases, the predicted extraction rates successfully maintain the pressure within the specified limits at the critical monitoring locations. These results demonstrate the applicability and robustness of the proposed workflow for managing pressure in a heterogeneous 3D multi-well reservoir system.

Finally, we evaluate the trained 3D multi-well model using 10,000 randomly generated permeability realizations. We use the predicted extraction rates from the model to run a full physics simulation and record the resulting pressures at both critical locations. The histograms in Fig. 11 shows that, for most of the cases, the pressures at critical locations remain close to the prescribed limits. The overall pressure deviation at the critical locations remains within approximately 0.2 kPa, confirming the capability of the workflow under complex realistic reservoir settings and geological uncertainty. The magnitude of the pressure variation is about an order lower than in the 2D case, which is consistent with the overall lower reservoir pressures resulting from the larger 3D domain.

## Discussion

In this study, we demonstrated that our pressure management workflow can accurately and efficiently manage pressure in heterogeneous subsurface reservoirs by controlling the fluid extraction rate. Similar approaches have previously been developed using simpler physics models, such as homogeneous permeability^40^ and single-phase flow^37^. We incorporate more complex physics into our model to represent a more realistic approximation of field operations. Our workflow uses a heterogeneous permeability fields and transient two-phase flow to improve prediction accuracy, while the use of automatic differentiation within the DPFEHM framework, and transfer learning enhances the efficiency of the model. This efficient and physics-based approach to pressure management will enable effective control of reservoir pressure and will aid in real-time decision making during injection and extraction operations in many subsurface applications, including gas storage, oil and gas production, wastewater disposal, and geothermal energy extraction.

### Advantages and scopes for improvement

The advantages of our workflow are as follows:

- Our model successfully predicts the extraction rate and maintains prescribed pressure at critical well locations.
- Due to the automatic differentiability in our background physics model, we are able to use a large number of ML parameters for training and testing.
- Our workflow uses a CNN model, which is fast and produces small errors.
- We create the training and testing datasets on the fly using the fully differentiable physics model and random realizations, rather than storing and loading data like many ML approaches.
- The large training data makes the model suitable for handling uncertainty in formation properties.
- Our workflow successfully uses transfer learning to reduce the high computational cost associated with complex transient two-phase flow by several orders of magnitude, which was identified as a major bottleneck in a past work^40^.
- By using transient two-phase flow, our model covers a wider range of applications than past work that used single-phase, steady-state flow and homogeneous properties^37,40^.

Although the presented workflow brings significant improvements over previous approaches and more accurately represents real-world complex physics, it still has some limitations and scope for improvement. Which are listed as follows:

- The current workflow uses simplified two and three-dimensional reservoir geometries, which are sufficient for this study as it serves primarily as a proof of concept. Future work should extend this framework to more realistic reservoir geometries that include faults, fractures, and folds, providing a closer representation of real-world CO$_2$ storage sites.
- The present study offers limited exploration of the effects of fluid properties, well configurations, and reservoir geometries on the learning process. Future research should systematically investigate how variations in these reservoir characteristics influence the performance, robustness, and transferability of the trained models.
- In this study we did not explore options to reduce the computational cost of training. Future study should focus on identifying the optimum number of random realizations required to achieve reliable model performance while minimizing computational cost. Additionally, optimization of grid resolution could be explored to balance numerical accuracy with efficiency.
- The background physics model was run for one year; however, gas storage operations typically span much longer timescales. A future study to extend the workflow should include simulation periods on the scale of years to more accurately represent field-scale pressure management.
- In our transfer learning workflow, the transition from the single phase steady state model to the more complex twophase model has been successful because the framework is physics-informed, and both stages share a similar setup and objective. However, in many applications, transfer learning may lead to misleading convergence or bias, especially when the physics used in pretraining differs significantly from that in the finetuning stage. In such cases, thorough evaluation using out of distribution parameters and appropriate benchmarking is essential to ensure accurate and reliable learning.

**Scalability and practical feasibility :** While the presented example in this study uses synthetic reservoir domains, the proposed workflow is designed to be directly extensible to realistic field-scale applications. The differentiable physics solver implemented here already supports 3D unstructured grids, which allows it to represent complex geological features such as faults, fractures, and folds. This capability enables seamless adaptation of the workflow to more detailed geological models used in real-field reservoir studies. Moreover, the solver is parallelized through MPI, which provides the computational scalability needed to handle the larger domain and longer simulation times required in real-field scenarios.

**Comparative baselines and data requirements:** As discussed in the introduction, integrating a physics solver directly into the training workflow enables more accurate, physics-informed learning. In this study, we did not compare our approach against other surrogate modeling methods because that will require labeled pairs (permeability and optimal extraction rate). For the problem we solve in this study, these labels are not available: the “optimal” rate is defined implicitly by the physics-constrained objective and would have to be computed by solving a per-sample optimization using the full simulator. Generating such a dataset on a scale (thousands of realizations) is the cost we seek to avoid. This motivated our physics-in-the-loop formulation, which learns the control policy without labels by evaluating the objective through the simulator during training.

**Choice of numerical method:** In this study, we successfully employed the IMPES method to demonstrate how a complex physics solver can be incorporated into a machine-learning workflow for reservoir pressure management. However, IMPES may face challenges when dealing with systems exhibiting strong nonlinearities and stiffness. Moreover, for long-term simulations in highly heterogeneous domains, fully implicit methods are generally more stable, efficient and are typically preferred. Incorporating such methods into our proposed workflow is straightforward, as the workflow developed here provides a solid foundation for such extensions.

Overall, very few studies in literature have applied differentiable programming for physics-informed ML application in reservoir pressure management. All the existing studies are limited to simplified physics, such as two dimensional steady state flow, making them primarily proof-of-concept demonstrations. Some of these works acknowledge the challenge of incorporating complex full-physics simulators into machine learning training^40^. However, no practical solution has yet been presented for integrating realistic complex reservoir physics into the learning process. Our study addresses this bottleneck by enabling the incorporation of a complex, transient two phase flow solver with a 3D multi-well reservoir setting into a differentiable machine learning framework. This development introduces an efficient and scalable approach for training surrogate models for reservoir pressure management in field scale applications

## Conclusions

We have introduced a novel pressure management workflow that integrates a fully differentiable, transient two-phase reservoir simulator with a CNN. Unlike previous works that rely on homogeneous properties or steady-state assumptions, our approach incorporates heterogeneous permeability fields and time-dependent two-phase flow, enabling high-fidelity pressure control at critical well locations.

One of the key strengths of this workflow is that by employing transfer learning from steady-state single-phase simulations to more complex two-phase simulations, we dramatically reduce computational cost while producing a highly accurate model. Another advantage of our workflow is its ability to generate large number of random training and testing data on the fly to effectively capture subsurface uncertainty. The use of automatic differentiation within the DPFEHM framework allows us to integrate of complex physics into the training process.

When evaluated across a wide range of randomly generated permeability fields, the workflow consistently predicts near-optimal extraction rates and maintains target pressures. The proposed approach delivers results that are not only accurate but also significantly faster than those produced by full-physics simulators at inference time, making it well-suited for near real-time applications. This workflow represents a meaningful step toward robust, efficient, and accurate pressure management across a wide range of real-world reservoir operations.

## Data Availability

The datasets generated and/or analysed during the current study are available in the [DiffMultiPhaseflowPresMngmnt] repository, [DiffMultiPhaseflowPresMngmnt].
